# Obstetrical outcome in pregnant women presenting with congenital uterine anomalies

**DOI:** 10.12669/pjms.41.4.10793

**Published:** 2025-04

**Authors:** Shahida Sutan, Samina Alia Sabir, Nazia Liaqat, Qudsia Qazi

**Affiliations:** 1 Shahida Sutan, FCPS Assistant Professor, Department of Gynae, Medical Teaching Hospital, Lady Reading Hospital, Peshawar Pakistan; 2 Samina Alia Sabir, FCPS Assistant Professor, Department of Gynae, Medical Teaching Hospital, Lady Reading Hospital, Peshawar Pakistan; 3 Nazia Liaqat, FCPS Associate Professor, Department of Gynae, Medical Teaching Hospital, Lady Reading Hospital, Peshawar Pakistan; 4 Qudsia Qazi, FCPS Associate Professor, Department of Gynae, Medical Teaching Hospital, Lady Reading Hospital, Peshawar Pakistan

**Keywords:** Congenital uterine abnormalities, Obstetric outcomes, Pregnancy

## Abstract

**Objective:**

To analyze the obstetrical outcome in pregnant women who presented with congenital uterine anomalies.

**Methods:**

Sixty patients participated in this descriptive study conducted at department of obstetrics and gynecology, Lady Reading Hospital, Peshawar from February 2023 to April 2024. Pregnant patients with CUA aged 20 to 35 years and gestational age 30 to 42 with singleton pregnancy were included. Classification of CUA was assessed along with negative obstetric outcomes. Data analysis was done using SPSS 20.

**Results:**

Septate uterus was detected in 13 (21.7%), unicornuate 5 (8.3%), bicornuate 32 (53.3%), and didelphys 10 (16.7%). Cesarean section 40 (66.7%), preterm delivery 21 (35%), malpresentation 33 (55%), SGA 36 (43.3%), and low birth weight 18 (30%) were negative obstetric outcomes. Unicornuate uterus and bicornuate uterus had greater caesarean section rates. Patients with unicornuate uterus, didelphys and septate had more preterm deliveries. Fetus malpresentation was higher in bicornuate uterus than other CUAs. Septate uterine patients had higher SGA and low birth weight rates.

**Conclusion:**

Our investigation confirms unfavorable obstetric outcomes are commonly linked to congenital uterine abnormalities.

## INTRODUCTION

Congenital uterine abnormalities (CUA) occur when there is a failure or partial development, canalization of either of the Mullerian ducts in fetal development.^1^ These defects have been linked to an increased risk of miscarriage, premature delivery, and other negative effects for the fetus.^2^ They are commonly observed as benign disorder with a broad range of occurrence. The exact prevalence of uterine abnormalities in the population remains undetermined.^3^ Consulting older medical literature is inadequate due to the inconsistent diagnostic approaches employed in earlier research and the variability of the subject populations that were examined.^3^

The proposed classification consists of six classes, which include normal uterus, dysmorphic uterus, septate uterus, unilaterally formed uterus, aplastic or dysplastic uterus, and unidentified malformation.^4^ The coexistent subclasses consist of cervical and vaginal abnormalities. Cervical anomalies can be classified in to typical, septate, and unilateral aplasia, and dysplasia.^4^ Vaginal abnormalities encompass several conditions, such as a typical vagina, a longitudinal non-obstructing vaginal septum, a longitudinal obstructing vaginal septum, and imperforate hymen.^4^

According to reports, uterine anomalies have been linked to a possible decrease in fertility and an increased risk of miscarriage.^5^,^6^ Uterine malformations can be categorized based on abnormalities in the embryological development process. These include defects in the unification of the Mullerian ducts, defects in the canalization process resulting in insufficient resorption for the midline septum, Mullerian agenesis, and arcuate uterus.^7^,^8^ Research findings indicate that the suggested abnormalities occur in 1-10% of the general population, 2-8% of women who are unable to conceive, and 5-30% of women who suffered a miscarriage.^9^

In addition, uterine abnormalities are frequently asymptomatic and incidentally detected during ultrasounds for additional gynecological conditions, evaluation of tubal patency, or pregnancy.^10^,^11^ In addition, they can also be identified during delivery, whether it is a spontaneous birth or a cesarean section.^12^ Congenital uterine abnormalities are a potential cause of small-for-gestational-age children, fetal malpresentation, recurrent pregnancy loss, and infertility; the effects are more pronounced in women with more serious abnormalities.^13^

Understanding obstetrical outcomes, advancing research and knowledge in the field of feto-maternal medicine are the main reasons for studying obstetrical outcomes in women with congenital uterine anomalies. Healthcare professionals can give patients experiencing uterine abnormalities with individualized, evidence-based care by looking into and assessing obstetrical outcomes. This enhances maternal and newborn health outcomes and improves patient-centered care. Hence we conducted this study to determine the obstetrical outcome in pregnant women presented with congenital uterine anomalies.

## METHODS

The current descriptive study was initiated at department of Obstetrics and Gynecology department of Lady Reading Hospital, Peshawar, Pakistan from February 2023 to April 2024.

### Ethical Approval:

The hospital ethics committee approved the study with Ref. No.: 139/LRH/MTI; dated May 6, 2024. Patients in the study were selected using non probability consecutive sampling.

**Table-I:** Distribution of obstetrical outcomes.

Obstetrical outcomes		N	%
Caesarean section	Yes	40	66.7%
	No	20	33.3%
Gestational age at delivery	Preterm	21	35.0%
	Term	33	55.0%
	Post term	6	10.0%
Malpresentation	Yes	33	55.0%
	No	27	45.0%
Small for Gestational Age	Yes	26	43.3%
	No	34	56.7%
Low birth weight	Yes	18	30.0%
	No	42	70.0%

### Inclusion criterion:

Pregnant women aged 20 to 35 years having singleton pregnancy presenting with gestational age 30 to 42 weeks diagnosed with CUA on ultrasonography (Toshiba Famio 5 ultrasonography device). Patients with multiple pregnancies or anomalies other than CUA were not included. The ultrasonography was performed by a radiologist having experience of more than five years. The uterine malformations were categorized using a specific and comprehensive system called the adapted ASRM classification system to obtain a more accurate representation of the prevalence of uterine anomalies.^4^

According to this system, uterine anomalies include uterus didelphys (having two external uterine orifices as well as two uterine bodies), bicornuate uterus (with a normal caudal part of the uterus and a bifurcated cranial part), complete or incomplete septate uterus (having a normal external surface, two uterine cavities, and one or two external uterine orifices), arcuate uterus (having a concave dimple in the uterine fundus inside the cavity), and unicornuate uterus (having one uterine endometrial cavity shifted to the right or left side), respectively. We assessed the obstetrical outcomes and its relationship with the classification of CUA in our patients. The outcomes were caesarean section, preterm delivery (< 37 weeks), SGA, low birth weight (< 2500 g), and malpresentation of the fetus (Transverse, breech or cephalic). Sample was estimated using openepi, taking previous frequency of Unicornuate uterus 3.1%^14^, margin of error 4.4% along with confidence interval 95%, the result was 60. Data analysis procedure was carried out using SPSS 23. Chi Square test was utilized for assessing the relationship between categorical variables, keeping the value of P significant at < 0.05.

## RESULTS

Sixty patients were taken for this study with mean age 27.67±4.50 years (20 to 35 years) while mean gestational age analyzed with 37.40±3.14 weeks (30 to 42 weeks). Septate uterus was found in 13 (21.7%) patients, unicornuate 5 (8.3%), bicornuate 32 (53.3%) and didelphys uterus in 10 (16.7%) patients. Obstetric outcomes recorded were caesarean section 40 (66.7%), preterm delivery 21 (35%), malpresentation 33 (55%), SGA 36 (43.3%) and low birth weight 18 (30%).

The association of classification of CUA with obstetrical outcomes is shown in Table-II. Frequency of caesarean section was notably higher in patients with unicornuate uterus 4 (80%) and bicornuate uterus 26 (81.2%). Preterm delivery’s frequency was higher in patients with unicornuate uterus 3 (60%), didelphys uterus 6 (60%) and septate uterus 7 (53.3%). In bicornuate uterus malpresentation of fetus was notably higher than other variations of CUA. Higher frequency of SGA and low birth weight were observed in septate uterus patients 10 (76.9%) and 6 (46.2%).

**Fig.1:**
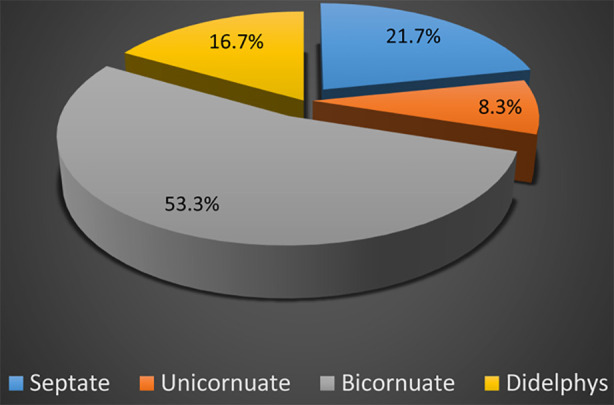
Frequency of classification of CUA.

**Table-II:** Association of classification of CUA with obstetrical outcomes.

Obstetrical outcomes		Various congenital anomalies								
		Septate		Unicornuate		Bicornuate		Didelphys		P-value
		N	%	N	%	N	%	N	%
Caesarean section	Yes	6	46.2%	4	80.0%	26	81.2%	4	40.0%	0.02
	No	7	53.8%	1	20.0%	6	18.8%	6	60.0%	
Gestational age at delivery	Preterm	7	53.8%	3	60.0%	5	15.6%	6	60.0%	0.02
	Term	6	46.2%	2	40.0%	21	65.6%	4	40.0%	
	Post term	0	0.0%	0	0.0%	6	18.8%	0	0.0%	
Malpresentation	Yes	7	53.8%	2	40.0%	22	68.8%	2	20.0%	0.04
	No	6	46.2%	3	60.0%	10	31.2%	8	80.0%	
Small for Gestational Age	Yes	10	76.9%	3	60.0%	10	31.2%	3	30.0%	0.02
	No	3	23.1%	2	40.0%	22	68.8%	7	70.0%	
Low birth weight	Yes	6	46.2%	2	40.0%	6	18.8%	4	40.0%	0.23
	No	7	53.8%	3	60.0%	26	81.2%	6	60.0%	

## DISCUSSION

When we analyze the anomalies we found that a large number of patients presented with bicornuate uterus 32 (53.3%) followed by septate various 13 (21.7%). Other anomalies observed in descending order were didelphys uterus accounting for 10 (16.7%) and unicornuate uterus accounting for 5 (8.3%). Several studies have reported that bicornuate uterus was the leading anomaly of CUA.^15^-^17^

A study reported that bicornuate uterus turned out to be a very frequent anomaly 43.8% out of five anomalies diagnosed in their patients.^14^ Another study also reported same pattern of CUA anomalies, they reported bicornuate was having the highest proportion 48% while unicornuate being the least present anomaly 3.1%, the slight elevating variation in unicornuate uterus in our study could be due to our larger sample size as compared to the aforementioned study, they enrolled 29 patients for their study while we had almost 50% larger sample size.^18^ Another study also reported similar pattern, bicornuate uterus in 55.1% patients, spectate in 19.1% patients, didelphys in 15.6% patients while unicornuate in 10.1% patients.^19^

These findings are in line with our observations about various classifications of CUA at presentation to our setup. Regarding the negative outcomes in our study, we observed that 40 (66.7%) patients had caesarean section, twenty-one patients (35%) were preterm at delivery, malpresentation was present in 33 (55%) patients while 26 (43.3%) newborns were small for their gestational age and 18 (30%) newborns had lower birth weight. Caesarean section has been reported in a study conducted on patients with CUA 63.5%, preterm delivery was reported as 25.7% for less than 37 weeks, nine percent for less than 34 weeks and 4.75% for less than 32 weeks. They reported birth weight < 2500 grams in 26.5% newborns.^19^

Another study reported small for gestational age in 37.93% newborns.^18^ These findings were similar to our observations. We ran a bivariate analysis of CUA classifications found in our patients with each negative outcome, we observed that caesarean section was frequently performed in patients with bicornuate and unicornuate anomalies (P = 0.02). A study reported that C-section rate in their study as higher in patients with unicornuate anomaly 82.4% followed by bicornuate anomaly 69.9%.^19^ We observed that preterm delivery was seen in 7 (53.8%) patients with septate uterus, in unicornuate 3 (60%), in bicornuate 5 (15.6%) and six (60%) in didelphys uterus. A systematic review reported from various studies that septate uterus and unification defects were substantially associated with preterm birth.^20^ We observed that fetal malpresentation was notably higher in bicornuate uterus 22 (68.8%) followed by septate uterus 7 (53.8%), unicornuate 2 (40%) and 2 (20%) in didelphys (P = 0.04). The aforementioned systematic review reported that patients with unicornuate and bicornuate uterus are at substantial risk for malpresentation.^20^

In our study newborns were small for their gestational age in 10 (76.9%) patients having septate uterus which was notably higher than other anomalies observed (P = 0.02) (Table-II), a study reported that in their patients SGA was notably higher in patients with canalization defect.^18^ We observed that low birth weight was recorded in 6 (46.2%) newborns born to patients having septate uterus, while the rate of low birth weight was higher in patients with unification defect, two (40%) in patients with unicornuate uterus, in patients with bicornuate uterus 6 (18.8%) and patients with didelphys uterus 4 (40%) however a notable difference was not observed (P = 0.23). Since previous studies conducted globally have highlighted CUAs as risk factors, our study quantifies how a specific anomaly correlate with negative outcomes, for illustration, it highlights that spectate uterus which is often considered as less severe has the highest rates for small for gestational age and preterm birth, thus challenging the previous assumptions about septate uterus being benign. Apart from negative obstetrical outcomes, we need further studies on how CUA can effect maternal mental health and wellbeing. We believe that this is the first study conducted in Khyber Pakhtunkhwa, Pakistan which has analyzed various classifications of CUA and its relationship with negative outcomes.

### Limitations:

It includes small sample of patients and not having a control group. We recommend further studies on CUA classification and its relationship with more negative outcomes across various centers.

## CONCLUSION

Our results confirm previous findings about the relationship of canalization and unification defects with negative outcomes. Congenital uterine anomalies are notably associated with negative obstetric outcomes.

### Author’s Contribution:

**SS:** Conception & design, data Collection, statistical analysis, prepared manuscript responsible for the accuracy of the work.

**SSabir:** Literature search, Compilation of results, preparing manuscript, and data collection.

**NL QQ:** Critical analysis, Reviewed the manuscript.

All authors have approved the final version.

## References

[1] Kang J, Qiao J (2024). Impact of congenital uterine anomalies on reproductive outcomes of IVF/ICSI-embryo transfer:a retrospective study. Eur J Med Res.

[2] Kim MA, Kim HS, Kim YH (2021). Reproductive, obstetric and neonatal outcomes in women with congenital uterine anomalies:a systematic review and meta-analysis. J Clin Med.

[3] Grimbizis GF, Campo R (2010). Congenital malformations of the female genital tract:the need for a new classification system. Fertil Steril.

[4] Grimbizis GF, Gordts S, Di Spiezio Sardo A, Brucker S, De Angelis C, Gergolet M (2013). The ESHRE/ESGE consensus on the classification of female genital tract congenital anomalies. Hum Reprod.

[5] Shahzad H, John A, Ali A, Ashraf A, Naeem MA (2022). Incidence of infertility in females and Evaluation of its Causes Using Ultrasonography:Incidence and Causes of Infertility in Females. Pak Bio Med J.

[6] Turocy JM, Rackow BW (2019). Uterine factor in recurrent pregnancy loss. Perinatology.

[7] Rackow BW Congenital uterine anomalies. Ultrasound Imaging in Reproductive Medicine. Adv Infertil.

[8] Kougioumtsidou A, Mikos T, Grimbizis GF, Karavida A, Theodoridis TD, Sotiriadis A (2019). Three-dimensional ultrasound in the diagnosis and the classification of congenital uterine anomalies using the ESHRE/ESGE classification:a diagnostic accuracy study. Obstet Gynecol.

[9] Lovelace D (2016). Congenital uterine anomalies and uterine rupture. J Midwifery Womens Health.

[10] Troiano RN, McCarthy SM (2004). Mullerian duct anomalies:imaging and clinical issues. Radiology.

[11] Ludwin A, Coelho Neto MA, Ludwin I, Nastri CO, Costa W, Acien M (2020). Congenital Uterine Malformation by Experts (CUME):diagnostic criteria for T-shaped uterus. Ultrasound Obstet Gynecol.

[12] Sugi MD, Penna R, Jha P, Pōder L, Behr SC, Courtier J (2021). Mullerian duct anomalies:role in fertility and pregnancy. Radiographics.

[13] Chan YY, Jayaprakasan K, Zamora J, Thornton JG, Raine-Fenning N, Coomarasamy A (2011). The prevalence of congenital uterine anomalies in unselected and high-risk populations:a systematic review. Hum Reprod update.

[14] Liston P, Gomathy E (2017). Obstetrical outcome in women with congenital uterine anomalies. Int J Reprod Contracept Obstet Gynecol.

[15] Cahen-Peretz A, Sheiner E, Friger M, Walfisch A (2019). The association between Mullerian anomalies and perinatal outcome. J Matern-Fetal Neonatal Med.

[16] Hiersch L, Yeoshoua E, Miremberg H, Krissi H, Aviram A, Yogev Y (2016). The association between Mullerian anomalies and short-term pregnancy outcome. J Matern Fetal Neonatal Med.

[17] Hua M, Odibo AO, Longman RE, Macones GA, Roehl KA, Cahill AG (2011). Congenital uterine anomalies and adverse pregnancy outcomes. Am J Obstet Gynecol.

[18] Elisa Zambrotta, Luisa Maria Di Gregorio, Federica Di Guardo, Roberta Agliozzo, Giuliana Chiara Maugeri, Ferdinando Antonio Gulino, Silvi (2021). Congenital uterine anomalies and perinatal outcomes:a retrospective single-center cohort study. Clin Exp Obstet Gynecol.

[19] Naeh A, Sigal E, Barda S, Hallak M, Gabbay-Benziv R (2022). The association between congenital uterine anomalies and perinatal outcomes - does type of defect matters?. J Matern Fetal Neonatal Med.

[20] Chan YY, Jayaprakasan K, Tan A, Thornton JG, Coomarasamy A, Raine-Fenning NJ (2011). Reproductive outcomes in women with congenital uterine anomalies:a systematic review. Ultrasound Obstet Gynecol.

